# Calendula as a Styptic, Etc.

**Published:** 1887-06

**Authors:** 


### Calendula as a Styptic, etc.

Marigold is a very valuable plant to dentists. A tincture (1 in 10) may be advantageously employed as a styptic to bleeding, lacerated, or granulating gums. It checks ulceration, and being far from nauseous to the taste, may be employed as a mouth wash, one part of the tincture to seven parts of water. If needful, a few drops of carbolic acid or some boracic acid may be added to the mouth wash. Dilute solutions are valuable in treating foul breath. It is said, also, that calendula is useful in treating the pockets in pyorrhœa alveolaris, after scraping; if so used, chloride of zinc (40 grains to ounce) should be used, together with the calendula. Calendula enjoys some reputation in the treatment of irritable pulp.

